# Novel approach to three-dimensional intermaxillary skeletal assessment: Correlation between the ANB angle and the bisector Wits appraisal

**DOI:** 10.1007/s00056-025-00583-0

**Published:** 2025-03-31

**Authors:** M. Serafin, B. Baldini, O. Rossi, G. Perrotti, A. Caprioglio

**Affiliations:** 1 Department of Biomedical Sciences for Health, University of Milan, Milan, Italy; 2 Department of Electronics, Information and Bioengineering, Politecnico Di Milano, Milan, Italy; 3 Department of Biomedical Surgical and Dental Sciences, University of Milan, Via della Commenda 31, 20122 Milan, Italy; 4 Fondazione IRCCS Cà Granda, Ospedale Maggiore Policlinico, Milan, Italy; 5 Private Practice, Como, Italy

**Keywords:** Three-dimensional cephalometry, ANB angle, Bisector-Wits, Cone-beam computed tomography, Wits appraisal, Dreidimensionale Kephalometrie, ANB-Winkel, „Bisector-Wits“, Digitale Volumentomographie, Wits-Beurteilung

## Abstract

**Purpose:**

The present study aimed to compare and correlate the ANB angle with the bisector Wits appraisal for a three-dimensional (3D) assessment of the maxillomandibular sagittal relationship using a cone beam computed tomography (CBCT) dataset.

**Methods:**

After outlier removal, 351 CBCT scans were chosen based on inclusion criteria (high quality, full-cranium field of view [FOV], slice thickness 150–300 μm) and analyzed using 3DSlicer software (Brigham and Women’s Hospital, Boston, MA, USA, version 5.2.2). Eight anatomical landmarks were manually annotated, identified on axial views, and confirmed on the rendered volume image. The coordinates of each landmark were exported and the ANB (°) and bisector–Wits (mm) measurements were constructed. Dahlberg’s D tested the intraobserver reliability and two-sample Kolmogorov–Smirnov test was executed to assess normality and to select the subsequent tests. Spearman’s rank correlation coefficient (ρ) was utilized to correlate the angular (ANB) and linear (bisector–Wits) measurements, whereas the Siegel estimator for nonparametric linear regression was employed to establish norm values by the correlation equation. Significance was set at *p* < 0.05 with correlation coefficients exceeding ρ > 0.70 deemed clinically relevant.

**Results:**

High (ρ = 0.773) and statistically significant (*p* < 0.001) correlations between the ANB and bisector–Wits measurements were found. The obtained equation was the following: bisector–Wits = 1.06 × ANB – 6.32. Therefore, the obtained rounded norm range for bisector–Wits for skeletal class I sagittal relationship was determined to be from −6.3 to −2.1 mm (−4.2 ± 2.1 mm). Values less than −6.3 mm correspond to a class III, whereas greater than −2.1 mm correspond to a class II skeletal relationship.

**Conclusion:**

The study revealed a statistically significant correlation between the ANB and bisector–Wits. From a 3D perspective, the bisector–Wits represents a reliable parameter to assess maxilla–mandibular skeletal discrepancies instead of the ANB angle, also adhering to radioprotection principles by limiting the FOV to the maxillary complex only and potentially reducing the radiation exposure in CBCT-based cephalometry.

## Abstract (de)

**Zielsetzung:**

Ziel der vorliegenden Studie war der Vergleich und die Korrelation des ANB-Winkels mit der Beurteilung des „bisector-Wits“ zum dreidimensionalen (3D) Assessment der sagittalen maxillomandibulären Beziehung unter Verwendung eines DVT(digitale Volumentomographie)-Datensatzes.

**Methoden:**

Nach dem Ausschluss von Ausreißern wurden 351 DVT-Aufnahmen auf der Grundlage der Einschlusskriterien (hohe Qualität, vollständiges Kranium-Sichtfeld [FOV], Schichtdicke 150–300 μm) ausgewählt und mit der Software 3DSlicer (Brigham and Womenʼs Hospital, Boston, MA, USA, Version 5.2.2) ausgewertet. Acht anatomische Orientierungspunkte wurden manuell beschriftet, auf axialen Ansichten identifiziert und auf dem gerenderten Volumenbild bestätigt. Die Koordinaten jedes Orientierungspunktes wurden exportiert, die ANB (°) und „bisector-Wits“ (mm) Messungen wurden konstruiert. Anhand von Dahlbergs D wurde die Intraobserver-Reliabilität getestet, zur Beurteilung der Normalität und zur Auswahl der nachfolgenden Tests wurde ein Kolmogorov-Smirnov-Test mit zwei Stichproben durchgeführt. Der Spearman-Rangkorrelationskoeffizient (ρ) wurde verwendet, um die Winkelmessungen (ANB) und die linearen Messungen („bisector-Wits“) zu korrelieren, während der Siegel-Schätzer für nichtparametrische lineare Regression verwendet wurde, um die Normwerte durch die Korrelationsgleichung zu bestimmen. Die Signifikanz wurde auf *p* < 0,05 festgelegt, und als klinisch relevant galten Korrelationskoeffizienten von mehr als ρ > 0,70.

**Ergebnisse:**

Zwischen den Messungen von ANB und „bisector-Wits“ zeigten sich große (ρ = 0,773) und statistisch signifikante (*p* < 0,001) Korrelationen. Die erhaltene Gleichung lautete: „bisector-Wits“ = 1,06 × ANB – 6,32. Daher wurde der gerundete Normbereich für „bisector-Wits“ für die sagittale Beziehung der skelettalen Klasse I auf –6,3 bis –2,1 mm (–4,2 ± 2,1 mm) festgelegt. Werte unter –6,3 mm entsprechen einer skelettalen Klasse-III-, Werte über –2,1 mm einer skelettalen Klasse-II-Beziehung.

**Schlussfolgerung:**

In der Studie wurde eine statistisch signifikante Korrelation zwischen ANB und „bisector-Wits“ festgestellt. Aus der 3D-Perspektive stellt der „bisector-Wits“ einen zuverlässigen Parameter zur Beurteilung von Diskrepanzen zwischen dem maxillaren und dem mandibulären Skelett anstelle des ANB-Winkels dar, der auch den Grundsätzen des Strahlenschutzes entspricht, indem er das FOV auf den Oberkieferkomplex begrenzt und die Strahlenbelastung bei der DVT-basierten Kephalometrie potenziell reduziert.

## Introduction

The ANB angle represents one of the most commonly used cephalometric parameters for assessing intermaxillary sagittal jaw relationships [1, 2]. However, many distorting factors are well known to affect the reliability of this angle: rotation of the jaws and/or occlusal plane with respect to the anterior cranial base, anteroposterior spatial relationship of nasion (N) with respect to the maxilla (A) and mandible (B), vertical dimension (distance from N to the upper molars) and increase in dental height (distance between A and B) [3–5]. These influencing factors affecting the cephalometric measurements were initially addressed by Panagiotidis and Witt in a 2-dimensional (2D) prospective [6].

In 1975, Jacobson introduced the Wits appraisal as a diagnostic aid, whereby the sagittal relationship between maxilla and mandible could be measured on a lateral cephalometric radiograph taking into account the occlusal plane. The Wits appraisal is determined by measuring the distance between the orthogonal projections of A and B points onto the occlusal which is drawn through the region of maximum cuspal interdigitation [7, 8].

Combining skeletal and dental landmarks, the Wits appraisal integrates both these components into its measurement and represents a valuable tool for assessing malocclusions that involve both dental and skeletal discrepancies, excluding extramaxillary variables that may influence the ANB angle [9, 10]. Nevertheless, if any dental compensation exists in the malocclusion, such as that by inclination of the incisors or vertical positioning of the molars that vary the cant of occlusal plane, the Wits appraisal is strongly influenced and it may not accurately reflect the true skeletal relationship [11, 12]. In order to overcome the limits of the traditional Wits appraisal of Jacobson, in 1994 Hall–Scott proposed a novel reference plane for the anteroposterior measurement of the dental bases, which involves bisecting the angle formed by the nasal line (NL) and the mandibular plane and he defined the parameter bisector Wits appraisal [12].

With the advent of cone beam computed tomography (CBCT), the three-dimensional analysis of skeletal structures has revolutionized the field of craniofacial imaging, offering clinicians unprecedented insight into the intermaxillary relationship [13, 14]. CBCT has attracted significant attention from practitioners seeking to enhance diagnosis and treatment for their patients; however, it is important to carefully evaluate the risks and benefits of CBCT on a case-by-case basis [15, 16]. Where and whenever possible, it is important and mandatory to minimize the radiation dose and reduce the field of view (FOV) of CBCT, especially in pediatric patients [17]. While the ANB angle involves extramaxillary structures especially in the area of the cranial base, the Wits or bisector Wits only requires intramaxillary reference points; therefore, a reduced FOV focusing only on maxillary regions is needed, minimizing unnecessary radiation exposure and optimizing imaging efficiency, maintaining the possibility of a 3D skeletal assessment based on the Wits or bisector Wits appraisals [18].

Therefore, the aim of the present study was to compare the ANB angle with the bisector Wits appraisal for a 3D assessment of the maxillomandibular sagittal relationship using a CBCT dataset.

## Materials and methods

This correlation study involved the analysis of a dataset comprising 354 CBCT scans obtained from orthodontic and maxillofacial patients orthodontically treated at the Department of Orthodontics and Maxillofacial Surgery of the IRCCS Fondazione Ca’ Granda Ospedale Maggiore Policlinico in Milan, Italy. The CBCTs used were obtained from the database of those patients who had previously received such a diagnostic procedure because of different reasons: impacted teeth like canines or third molars, agenesis or supernumerary teeth, all without obvious skeletal asymmetries. No patient was scanned solely for the purpose of the present study. The retrospective study protocol underwent a thorough review and received approval from the Ethical Committee of the University of Milan (protocol number 71/22, dated 21 July 2022). The procedures strictly adhered to the principles outlined in the World Medical Organization Declaration of Helsinki. Prior to any treatment, informed consent for scientific purposes was obtained from all participants involved in the study.

All CBCT scans were systematically chosen based on the specified inclusion criteria, which included good quality scans, a full-cranium FOV including all landmarks necessary for the study, and a slice thickness ranging between 150–300 μm. Patients with severe asymmetry, a history of previous orthognathic treatment as well as those with systemic diseases or syndromes were excluded from participation.

The DICOM files from each patient underwent analysis and were imported into a dedicated software (3DSlicer, Brigham and Women’s Hospital, Boston, MA, USA, version 5.2.2) for the manual annotation of a total of 8 landmarks (7 monolateral, 1 bilateral). Each landmark was identified on 2D axial views, and its position was subsequently verified on the 3D rendered volume image. The list of landmarks and their definitions are reported in Table 1.

**Table 1:** List of landmarks and their definitions Liste der Orientierungspunkte und ihre Definitionen

Landmark	Abbreviation	Definition
Nasion	N	The most anterior aspect of the frontonasal suture, observed on the median sagittal plane
Sella	S	Three-dimensional center of the sella turcica
A point	A	The most posterior aspect of the concavity between the ANS and the alveolar bone of the upper incisors, observed on the median sagittal plane
B point	B	The most posterior aspect of the concavity between the pogonion and the alveolar bone of the lower incisors, observed on the median sagittal plane
Anterior nasal spine	ANS	The most anterior aspect of the nasal spine, observed on the median sagittal plane
Posterior nasal spine	PNS	The most posterior aspect of the nasal spine, observed on the median sagittal plane
Gonion	Go	The most posteroinferior point of the gonial angle, observed on the right and left sides of the mandible
Menton	Me	The lowest aspect of the mandibular symphysis, observed on the median sagittal plane

After the manual landmarking, the coordinates from each reference point were exported and analyzed through a programming language (Python, version 3.11.4). Therefore, the ANB (°) and bisector–Wits (mm) measurements were constructed. The ANB angle was determined as a difference between two angles following the formula:

$$ANB=SNA-SNB$$

**Fig. 1:**
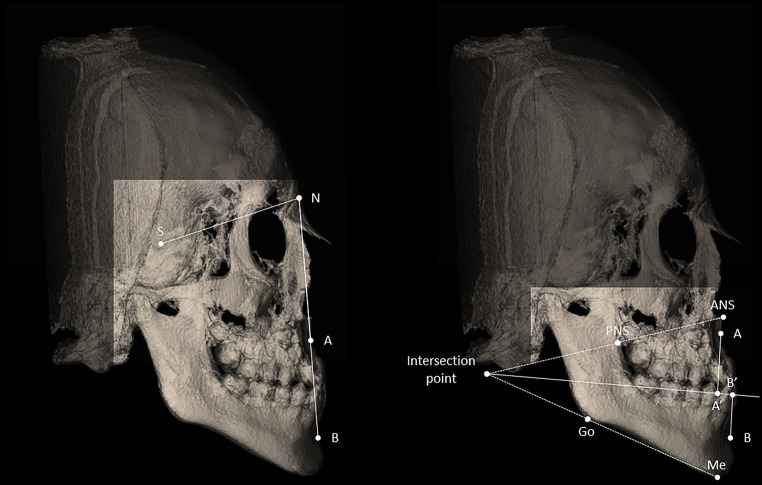
Cephalometric template representing the construction between angular ANB (left) and linear bisector–Wits (right) measurements. Transparent areas underline the difference in the required field of view (FOV) between the two techniques Kephalometrische Schablone zur Darstellung der Konstruktion zwischen angulären Messungen, ANB (links), und linearen Messungen, „bisector-Wits“ (rechts). Transparente Bereiche unterstreichen den Unterschied im erforderlichen Blickfeld (FOV) zwischen den beiden Techniken

The bisector–Wits measurement required a preparatory step: first, a mandibular plane (MP) passing through right and left gonion, and menton landmarks was constructed; then, the intersection point between MP and the NL line, passing through ANS-PNS, was determined. The bisector plane between the MP and the NL line, passing through the intersection point, was calculated. Finally, the orthogonal projections of A (A’) and B (B’) points onto the bisector plane were used to calculate the distance between these points to the intersection point. The bisector–Wits was obtained as the difference between the distances following the formula:

$$\begin{aligned}\textit{Bisector}-Wits={}&d\left(A^{\prime},\textit{intersection}\,\textit{point}\right)-\\&d\left(B^{\prime},\textit{intersection}\,\textit{point}\right)\end{aligned}$$

where d (x, y) represents the Euclidean distance between two points. Figure 1 provides a graphical representation of the measurement.

For the calculation of the error of the method and the intraobserver reliability for the tested variables Dahlberg’s formula was used:

$$\textit{Method}\,\textit{error}=Var(d_{i})=\Sigma d_{i}^{2}/N$$

where *d*_*i*_ is the difference between the first and the second measurements and *N* is the number of paired observations. A total of 25 randomly selected CBCT scans were annotated twice by the same trained operator and the results were analyzed for the estimation of the Dahlberg D. The established values ranged from 0.1 to 1.1° for angular and from 0.2 to 0.6 mm for linear measurements. Based on these results, a low degree of variability was obtained and therefore the measurements were considered reliable for further analyses.

Statistical analysis was performed using Python. First, data were tested for normality by using two-sample Kolmogorov–Smirnov test with approximation method and α = 0.05 to select the appropriate parametric or nonparametric test. Since the two samples did not show normal distributions, nonparametric tests were selected. Outlier measurements were detected using the Tukey’s fences method based on the interquartile range (IQR), where outliers are typically defined as data points that lie below the first quartile (Q1) and the third quartile (Q3) of the data multiplied by a constant. In this study, the range was defined as

$$IQR=[Q1-1.5\times IQR;Q3+1.5\times IQR]$$

The inferential statistical analysis included Spearman’s correlation coefficient (ρ) to assess the strength and direction of the relationship between the angular measurement ANB (independent variable) and the linear measurement bisector–Wits (dependent variable). Levels of *p* < 0.05 were considered statistically significant and correlation coefficients greater that ρ > 0.70 were considered strong and clinically relevant. Finally, Siegel estimator for nonparametric linear regression tested the relationship between the variables.

Since the NL^MP angle (skeletal vertical relationship) forms part of the purposed method and to further analyze its influence on the relationship between the ANB and bisector–Wits, a multivariate regression analysis was performed. This additional regression was conducted to assess whether incorporating those variables into the model would significantly improve the prediction of the bisector–Wits.

Finally, the interrater agreement between the two methods was evaluated by Cohen’s kappa statistic (κ) and contingency matrix based on the present dataset.

## Results

The correlation between the ANB angle and the bisector–Wits was tested on 354 CBCT scans consecutively selected. Outlier detection highlighted 3 cases which were removed from the dataset to standardize the statistical calculations. Therefore, correlations and linear regressions were performed on 351 cases (212 males, 139 females; mean age 18.4 ± 8.1 years, range 6.4–48.2 years). The sample was characterized by a mean ANB value of 3.88 ± 2.78° and a mean bisector–Wits value of −2.23 ± 4.26 mm.

To provide new clinical norm ranges for the measurements obtained by the bisector–Wits, first the strength and direction of the relationship were evaluated by nonparametric Spearman correlation coefficient [19–21]. A high (ρ = 0.773) and statistically significant (*p* < 0.001) correlation was found. This value (ρ > 0.70) was considered clinically relevant. The positive sign indicated that there was a positive monotonic relationship. Moreover, post hoc analysis applying the Siegel estimator for nonparametric linear regression was used to model the relationship between the two sets of measurements. The obtained equation was the following:

$$\textit{Bisector}-Wits=1.06\times ANB-6.32$$

**Fig. 2:**
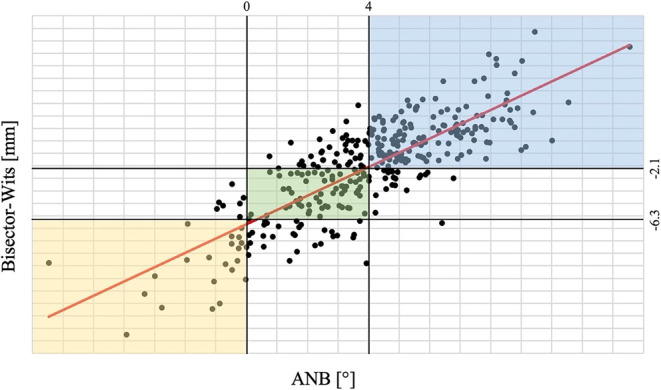
Siegel linear regression graph between independent (x, ANB) and dependent (y, bisector–Wits) variables. The green box represents the consistent and contingency ranges for skeletal class I measurements while the blue and the yellow boxes for skeletal class II and class III, respectively Lineare Regressionskurve nach Siegel zwischen unabhängigen (x, ANB) und abhängigen (y, „bisector-Wits“) Variablen. Die grüne Box stellt die konsistenten und die kontingenten Bereiche für die skelettalen Klasse-I-Messungen dar, die blauen und gelben Boxen die Klasse-II- bzw. -III-Messungen

Therefore, the rounded corresponding norm to classify the sagittal discrepancy as class I for the bisector–Wits compared to the ANB (2 ± 2°) was assessed to be −4.2 ± 2.1 mm. Thus, values less than −6.3 mm correspond to a class III relationship, whereas values greater than −2.1 mm correspond to a class II relationship. Figure 2 represents the linear regression between the included parameters.

In evaluating the robustness of the multivariate model, the initial simple linear regression model, where the ANB alone predicted the bisector–Wits, was compared with a multivariate regression analysis including the NL^MP angle. The initial model yielded a significant R‑squared value of 0.567, indicating that the ANB alone explained 56.7% of the variability of the bisector–Wits. When the NL^MP angle was added as a predictor, the R‑squared increased modestly to 0.639, with an adjusted R‑squared of 0.637, suggesting a slight improvement in model fit without overfitting. To confirm robustness, a robust regression using the Siegel estimator was conducted, yielding coefficients of −1.06 for the ANB, and −0.14 for the NL^MP angle, all with *p*-values below 0.001. These coefficients closely matched the initial model, demonstrating that adding NL^MP as a predictor did not compromise the reliability of the ANB in predicting the bisector–Wits.

The κ score between the ANB and bisector–Wits classifications was found to be 0.495. This result suggests a moderate level of agreement between the two methods, indicating that while the classifications are generally consistent, there are instances of discrepancy between them. Table 2 reports the cross tabulation between the ANB and bisector–Wits classifications.

**Table 2:** Contingency table regarding the classification of class I, II, and III cases between the ANB and bisector–Wits methods Kontingenztabelle zur Einteilung der Klasse-I-, -II- und -III-Fälle zwischen den Methoden: ANB und „bisector-Wits“

		Bisector–Wits			
		Class I	Class II	Class III	Total
*ANB*	*Class I*	67	43	28	138
	*Class II*	22	157	1	180
	*Class III*	8	0	25	33
–	Total	97	200	54	*351*

The highest concordance value was reached particularly in class II, followed by class I cases. Notably, except for one isolated case, there were no misclassifications between class II and class III, whereas the other misclassifications contributed to the moderate κ score.

## Discussion

The present correlation study explored the intricate relationship between the ANB angle and the bisector–Wits measurement from a three-dimensional perspective, examining the norm range values for the application of the bisector–Wits in a reduced or ultrareduced FOV CBCT-based cephalometric analysis. The following discussion tries to demonstrate the validity of this method with respect to the ANB angle.

The ANB and Wits appraisal have already been investigated from a 2D [6] and 3D perspective [18, 22] and, in appraising horizontal disharmony of the face and the anteroposterior jaw relationships, they are the most commonly used parameters, although the well-known limitations [12, 23]. Panagiotidis in 1977 [6] and Jacobson in 1979 underlined how a rotation of the jaws relative to the cranial base reference plane (SN) or a different anteroposterior position of the points nasion (N) or sella (S) decisively affect the ANB angle reading [9, 23]: when there is an increase in cranial divergence, the ANB angle tends to decrease and the mandible appears to be positioned relatively more forward in relation to the maxilla. A decreased skeletal divergence tends to result in an increased ANB angle and the mandible appears relatively retruded compared to the maxilla. Also, the position of nasion affects the orientation of the reference plane used for the ANB angle measurement: the ANB angle tends to be smaller when N is positioned more anteriorly, indicating a more forward position of the mandible relative to the maxilla. A more posteriorly positioned N results in a larger ANB angle, suggesting a retruded mandibular position. Moreover, the position of S is integral to the calculation of both the SNA and SNB angles, and variations in its position influence the ANB angle [3–6].

To transcend these constraints and eliminate variables beyond the treatment control, Jacobson introduced the Wits appraisal to measure the jaws anteroposterior relationship focusing on the lower third of the face, no longer taking into account extramaxillary references as nasion and sella but the occlusal plane. The Wits appraisal is less influenced by variations in craniofacial anatomy and skeletal landmarks and depends more on the position of the teeth [7, 8, 23]. However, detecting the occlusal plane in order to assess the Wits appraisal is sometimes difficult and controversial. Dental anomalies, missing teeth, mispositioned teeth, dental restorations, molar overlapping, dental anatomical variation, different tooth inclination, vertical movement or eruption to adapt to underlying skeletal discrepancies, the transition from the deciduous to the permanent dentition, and a pronounced curve of Spee influence the detection and the resulting cant of the occlusal plane and, thus, interpretation of the Wits appraisal [12].

Furthermore, different definitions of occlusal plane are cited in the literature: 1) the functional occlusal plane (FOP), a plane passing through the molar and premolar contacts [24], 2) the bisecting occlusal plane (BOP), a plane bisecting the overlap of the distobuccal cusps of the first permanent molars and the incisor overlap, 3) the lower incisor occlusal plane (LIP), and 4) the functional aesthetic occlusal plane (FAOP) [10, 25]. So many definitions make interpretation of the Witt appraisal difficult [26]. For the FOP, the reference points are lying very close together and this makes identification of the plane difficult to construct even under perfect conditions. Further confusion may be caused by shedding of deciduous molars before the eruption of the premolars, when the absence of the premolar point prevents definition of the FOP [12, 27]. In addition, the cant of the FOP changes with growth. This rotation happens in a random fashion during growth and is independent of the rotational growth pattern of the jaws. Thus, the Wits assessment may change throughout the growth period [28–30].

Hall-Scott introduced the maxillary–mandibular planes angle (MM) bisector as a new reference plane for the anteroposterior measurement to overcome the traditional Wits appraisal shortcomings, minimizing the impact of dental compensations by bisecting the NL line and the mandibular plane [12, 31]. The plane is a geometrical construct bisecting the angle formed by the MP (Go-Me) and NL plane (ANS-PNS), excluding any dental reference point from the measurement. This can be advantageous in cases where there are significant posterior dental compensations or changes in incisor inclination that may affect the cant of the occlusal plane and the accuracy of traditional Wits appraisal. Another strong supporting element for the use of the bisector–Wits is the ease in locating the reference points for its construction, especially when this is done on 3D radiological images [15, 31, 32].

Finally, using an intermediate plane between the maxilla and the mandible also minimizes factors related to the skeletal vertical dimension, such as excessive vertical maxillary growth or an elongated mandibular symphysis. Additionally, the identification of a single bisector plane provides a highly reliable measurement that accounts for potential deformations not only in the sagittal plane (pitch) but also in the coronal (roll) and axial (yaw) planes [3, 12].

Also, it should be noted that, as it is the case with the Wits appraisal, the bisector Wits appraisal is often discrepant with the ANB angle. Such inconsistencies underscore the importance of using multiple skeletal assessment methods but also include multiple variables in considering the skeletal classification. The performed multivariate regression analysis further illuminated these differences by assessing how additional variables, such as the skeletal vertical dimension, influenced the relationship between the ANB and the bisector Wits. Moreover, the moderate contingency values underscore that relying solely on the ANB or the bisector Wits may not capture the full scope of a skeletal discrepancy, particularly in complex cases. Despite that, since the ANB is based on structures far from the jaws whose configuration affects it, the bisector Wits appraisal, on the other hand, considers only the basal jaw bones as the refence and the derived skeletal classification might be considered more reliable than that based on the ANB.

Therefore, at the end of this discussion, it seems to become evident how the bisector–Wits approach can generate a wealth of patient-related information, free from potential dental influences, and enable a reduction of the radiological area of interest. Both the bisector–Wits and the traditional Wits of Jacobson focus on the lower third of the face, allowing for a more specific and limited assessment [7, 8, 12, 23]. The radiation protection principles necessitate adjusting the FOV to be consistent with the required information and to reduce exposure to ionizing radiation as much as possible [32]. With this approach, it is possible to determine the sagittal skeletal class of the maxillaries by performing CBCT scans with a reduced FOV, allowing for a three-dimensional view limited to the maxillary bones, while excluding areas of minor relevance to orthodontics, such as the neurocranium, or radiation-sensitive organs like the crystalline lens or the thyroid gland [13, 15].

## Limitations

One significant limitation of this study is that it focused largely on Caucasian patients. Therefore, the findings from this study may not be directly applicable or generalizable to individuals from other ethnic backgrounds. Moreover, there is a potential bias for unaccounted confounding variables. Factors such as genetics, functional influences, or environmental variables that were not specifically controlled for in the study might have influenced the observed correlation.

Additionally, the modest improvement in model fit after including the skeletal vertical dimension in the multivariate regression suggests that, while this additional variable enhanced predictive capability, the model’s ability to explain the variance in bisector–Wits remains moderate. This limitation implies that other unaccounted factors, such as divergency or individual anatomical variations, might influence skeletal relationships and thus affect the generalizability of the findings, as well as the agreement between the analyzed methods for skeletal assessment.

## Conclusion

This correlation study has provided valuable insights into the use of the bisector–Wits in 3D cephalometric skeletal assessment. The findings indicate a statistically significant correlation between the bisector–Wits linear measurements and the ANB angle. The norm value for the sagittal discrepancy identified by the bisector–Wits ranged from −6.3 to −2.1 mm to classify a skeletal class I; values less than −6.3 mm and greater than −2.1 mm correspond to a skeletal class III and class II relationship, respectively. Ultimately, integrating the bisector–Wits into routine assessments may pave the way for advancements in orthodontic practice while respecting radioprotection principles by limiting the field of view (FOV) and the subsequent radiation dose in cone-beam computed tomography (CBCT)-based cephalometry.

Future research should consider expanding the sample to include more diverse populations and exploring additional skeletal and dental parameters that may further improve diagnostic accuracy. Incorporating a multivariate approach with both intra- and extramaxillary factors could provide a more robust foundation for skeletal assessment and aid in achieving more individualized and precise treatment planning in orthodontics.

## Data Availability

In accordance with patient privacy regulations and ethical guidelines, the data supporting the findings of this study are not publicly available. However, they can be accessed upon reasonable request. Researchers seeking to obtain the data should contact the authors, providing a clear and justified purpose for the data request. All requests will be reviewed to ensure compliance with patient confidentiality and data protection policies.
